# Disaffection in the State Medical Association of Georgia

**Published:** 1878-05-20

**Authors:** 


### EDITORIAL AND MISCELLANEOUS.

### DISAFFECTION IN THE STATE MEDICAL ASSOCIATION OF GEORGIA.

All true men in the Medical Profession cannot but regret the frequent occurrence of divisions and difficulties amongst us. So important is harmony to the progress of medical science that little can be expected while such troubles exists in our ranks. We all admit that it is very desirable to avoid these troubles and to adjust and remove them where they exist. There are occasions when a little oil upon the troubled waters, or silence itself, with a little time, will adjust them; but there are other times when but little can be hoped from this method, and when it would seem to be the duty of every good and true man to enter his protest against the evils which exist, and which if unchecked, may eventuate in the ruin and professional disgrace of all concerned.

At the meeting of the Association, which was recently held in Atlanta, the proceedings of which we publish in the present number of our journal, the manifestations of discontent were unmistakable. These were evinced not so much in the official action of the body as in the quiet interviews and private conversations of its members.

Since the adjournment of the Association, we have received information and letters from different sections of the State expressing objections to its management.

Some of these are from gentlemen of prominence and ability. They claim that there are a large number of the members who are dissatisfied. To show the drift of these objections, we give a few of the points presented. It is claimed that The State Society is a migratory body, and not an association proper, and is mainly kept up by large initiation fees from local additions gathered at the different points at which it assembles. Many who thus join do not meet the Society again until after the lapse of years, when it returns to their vicinity, and perhaps never at all. In the meanwhile, notwithstanding the liberal initiation fee paid, the members are assessed annually, and duns sent out, accompanied by threats to drop from the roll if not responded to. That a favored few manage it by a caucus method to suit their own interests and views—confering the offices and honors, with but few exceptions—upon their special friends and favorities, rather than in a manner to represent impartially the entire membership.

These, and other similar objections, are urged, all of which might perhaps be remedied could a full meeting and free expression be had; but there is one other point which has been brought to our notice, and has sought expression through our pages, which bears a far more serious aspect, and that is that there are those in the association who have violated the ethics, and that the fact is well known and has been acquiesced in by the official action of the body itself, or at least by those who control it.

This much as journalists, we have deemed it proper to say in justice to the large number of those who make these complaints, and who claim that it is due to truth and to the honor of the medical profession of Georgia that these facts be made known, and, if possible, a remedy be found.

### GELATINE COATED PILLS—McKESSON & ROBBINS.

These elegant preparations, as prepared by the splendid establishment of McKesson & Robbins, manufacturing chemists, New York, are certainly very acceptable to the profession. We have received a number of beautiful samples of their preparations, and have tested them with much satisfaction. We regard them as eminently reliable and successful business men, and deserving of the confidence of the profession and the public.

